# Dietary delphinidin inhibits human colorectal cancer metastasis associating with upregulation of miR-204-3p and suppression of the integrin/FAK axis

**DOI:** 10.1038/s41598-019-55505-z

**Published:** 2019-12-12

**Authors:** Chi-Chou Huang, Chia-Hung Hung, Tung-Wei Hung, Yi-Chieh Lin, Chau-Jong Wang, Shao-Hsuan Kao

**Affiliations:** 1 Department of Colorectal Surgery, Chung Shan Medical University Hospital, Taichung, Taiwan; 2 School of Medicine, Chung Shan Medical University, Taichung, Taiwan; 3 Institute of Biochemistry, Microbiology, and Immunology, College of Medicine, Chung Shan Medical University, Taichung, Taiwan; 4 Institute of Medicine, Chung Shan Medical University, Taichung, Taiwan; 5 Division of Nephrology, Department of Internal Medicine, Chung Shan Medical University Hospital, Taichung, Taiwan; 6 Clinical Laboratory, Chung Shan Medical University Hospital, Taichung, 402 Taiwan

**Keywords:** Gastrointestinal cancer, Cell signalling

## Abstract

Delphinidin is a flavonoid belonging to dietary anthocyanidin family that has been reported to possess diverse anti-tumoral activities. However, the effects of delphinidin on colorectal cancer (CRC) cells and the underlying mechanisms are not fully understood. Thus, we aimed to investigate the anti-cancer activity of delphinidin in CRC cells and the underlying molecular mechanisms. The effects of delphinidin on the viability, metastatic characteristics, signaling, and microRNA (miR) profile of human CRC cell lines used were analyzed. *In vivo* metastasis was also evaluated using xenograft animal models. Our findings showed that delphinidin (<100 μM) inhibited the colony formation of DLD-1, SW480, and SW620 cells, but non-significantly affected cell viability. Delphinidin also suppressed the migratory ability and invasiveness of the tested CRC cell lines, downregulated integrin αV/β3 expression, inhibited focal adhesion kinase (FAK)/Src/paxillin signaling, and interfered with cytoskeletal construction. Analysis of the miR expression profile revealed a number of miRs, particularly miR-204-3p, that were significantly upregulated and downregulated by delphinidin. Abolishing the expression of one upregulated miR, miR-204-3p, with an antagomir restored delphinidin-mediated inhibition of cell migration and invasiveness in DLD-1 cells as well as the αV/β3-integrin/FAK/Src axis. Delphinidin also inhibited the lung metastasis of DLD-1 cells in the xenograft animal model. Collectively, these results indicate that the migration and invasion of CRC cells are inhibited by delphinidin, and the mechanism may involve the upregulation of miR-204-3p and consequent suppression of the αV/β3-integrin/FAK axis. These findings suggest that delphinidin exerts anti-metastatic effects in CRC cells by inhibiting integrin/FAK signaling and indicate that miR-204-3p may play an important role in CRC metastasis.

## Introduction

Colorectal cancer (CRC) is a leading cause of death in countries worldwide, including Taiwan, and it is the second and third most common cancer in women and men, respectively^1^. CRC originates from dysplastic adenomatous polyps that occasionally become cancerous, primarily due to genetic alterations that promote proliferation and inhibit apoptosis in colorectal epithelial cells^2^. Although chemotherapy, surgery, and novel anti-cancer drugs have been developed for the treatment of CRC, rapid and distant metastases lead to high mortality rates^3^. The acquisition of cell adhesion, migratory ability, and invasiveness plays a pivotal role in the metastasis of CRC^4^. Thus, these metastatic characteristics of CRC cells could be important treatment targets.

Cancer is a life-threatening malignant disease. Therefore, identifying molecules, including phytochemicals, with strong anti-tumoral potential and low toxicity is important for the development of effective treatments and novel drugs. Over the past decade, numerous natural compounds have been shown to possess chemopreventive and anti-tumoral activities *in vitro* or *in vivo*^5–8^, and accumulating evidence has shown that anthocyanins and anthocyanidins exhibit various biological activities, including anti-inflammatory^9^, anti-tumoral^10^, and anti-carcinogenic effects^11^. Delphinidin is the major anthocyanidin in many dietary fruits and vegetables, such as berries and tomatoes, and it has been shown to possess potent anti-oxidant, anti-inflammatory^12^, and anti-tumoral properties^13^.

MicroRNAs (miRs) are short (22–24 nucleotide) non-coding RNAs that bind to the 3′-untranslated region of coding RNAs by base pairing, which results in termination of protein translation or degradation of messenger RNA (mRNA)^14^. miRs have been implicated in embryonic development^15,16^, cellular homeostasis^17,18^, physiopathological conditions^19,20^, and dysregulated expression of oncogenic genes involved in various cancers, including renal cell carcinoma^21^, ovarian cancer^22^, and hepatocellular carcinoma^23^. miR dysregulation has also been shown to be involved in the induction of epithelial-mesenchymal transition (EMT) in various tumors^24,25^. This evidence indicates that uncontrolled miR expression may be associated with carcinogenesis, cancer progression, and tumor metastasis.

In this study, we aimed to investigate the effects of delphinidin on colon cancer metastasis, with an emphasis on the regulation of miR expression and integrin-associated signaling. Cell viability was assessed by the MTT assay. Carcinogenic properties were evaluated by adhesion and colony formation assays. Cell motility and invasiveness were assessed by transmigration and invasion assays. The related signaling cascades were evaluated by western blotting, and the miR expression profile was analyzed by microarray and quantitative real-time reverse transcription poly chain reaction (qRT-PCR).

## Results

### Delphinidin had non-significant effects on the viability of human CRC cells

**Figure 1:**
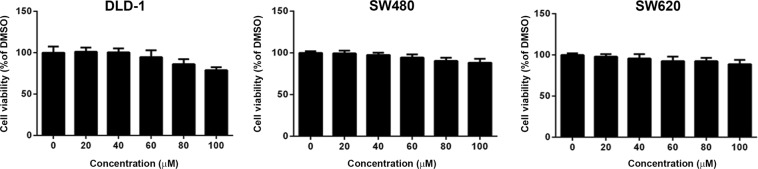
Effects of delphinidin on the viability of human CRC cells. Cells were treated with delphinidin at serial concentrations for 24 h, and then the cell viability was assessed by MTT assay. Cell viability was presented as percentage of the DMSO control (0 μM) in mean ± standard deviation. No significant difference was observed among each treatment.

To investigate the anti-tumoral effects of delphinidin in CRC, its effects on cell viability were first analyzed in three human CRC cell lines: DLD-1, SW480, and SW620. The results showed that delphinidin, at 20–100 μM, had non-significant effects on the viability of the three cell lines (Fig. 1, *P* > 0.05). These findings indicate that delphinidin does not influence the viability of CRC cells.

### Delphinidin diminished the colony formation and adhesion of human CRC cells

**Figure 2:**
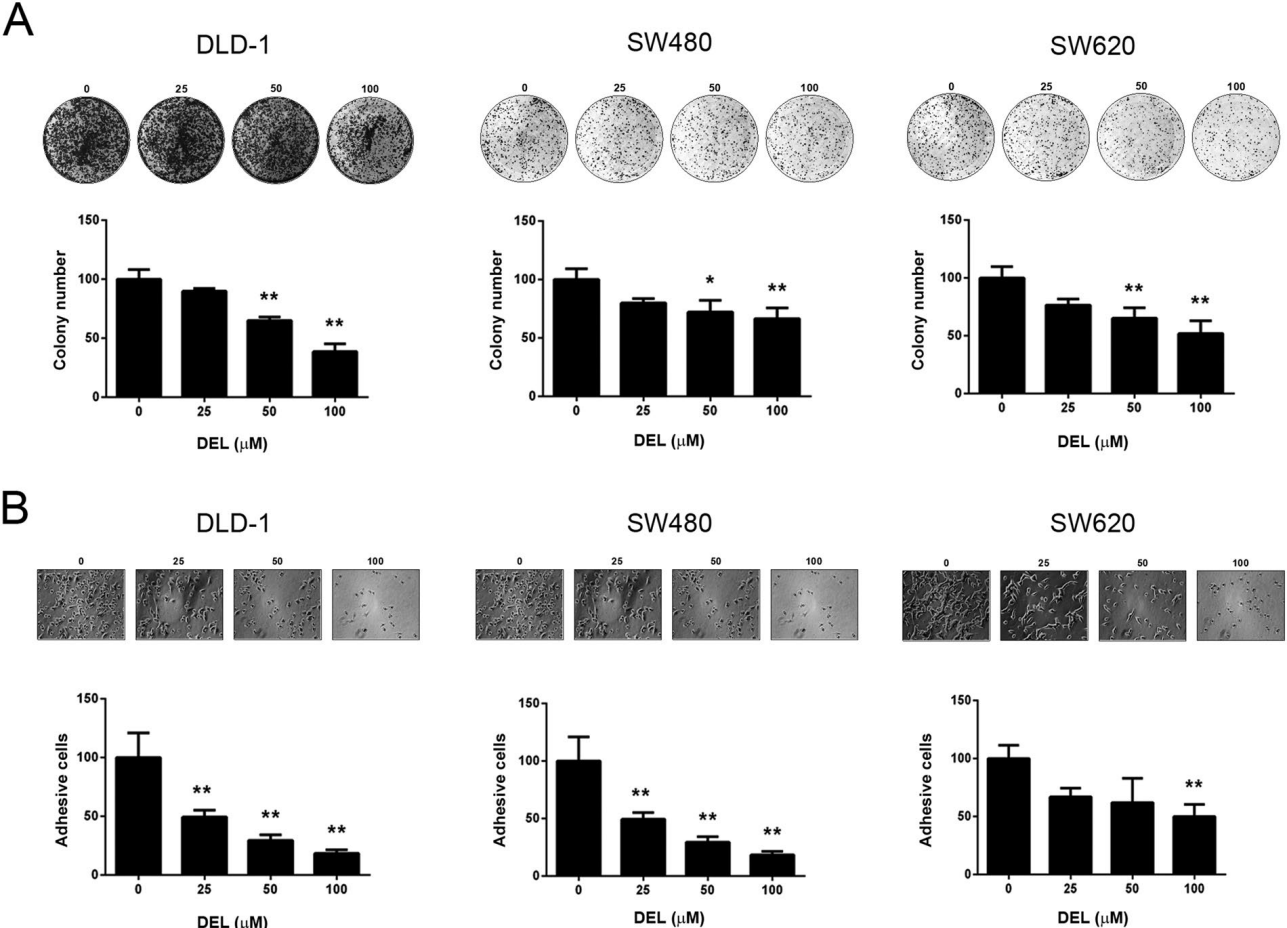
Delphinidin inhibited colony formation and cell adhesion of human CRC cells. Cells were cultured in soft agar or collagen-coated plates, treated with delphinidin (DEL) at serial concentrations for 24 h, and then subject to (A) colony formation assay or (B) cell adhesion assessment, respectively. Quantitative data was acquired from three independent experiments and presented as mean ± standard deviation. * and **, *P* < 0.05 and *P* < 0.01 as compared to the DMSO control (0 μM).

Colonization on soft gel and attachment to extracellular matrix components, such as collagen, are notable characteristics of malignant cancer cells^26^. Since the viability of CRC cells was not affected by delphinidin, we explored its effects on colony formation and cell adhesion. As shown in Fig. 2A, 50 and 100 μM delphinidin significantly (*P* < 0.01) and dose-dependently decreased the number of colonies formed on soft agar by DLD-1, SW480, and SW620 cells. Similarly, the number of cells that adhered to culture plates coated with type I-collagen was also dose-dependently decreased by delphinidin treatment (*P* < 0.01; Fig. 2B). Interestingly, the number of attached SW620 cells was only significantly reduced following treatment with 100 μM delphinidin (Fig. 2B). Collectively, these results show that delphinidin diminishes the colony formation and ECM adhesion ability of CRC cells.

### Delphinidin inhibited the migration and invasion of human CRC cells

**Figure 3:**
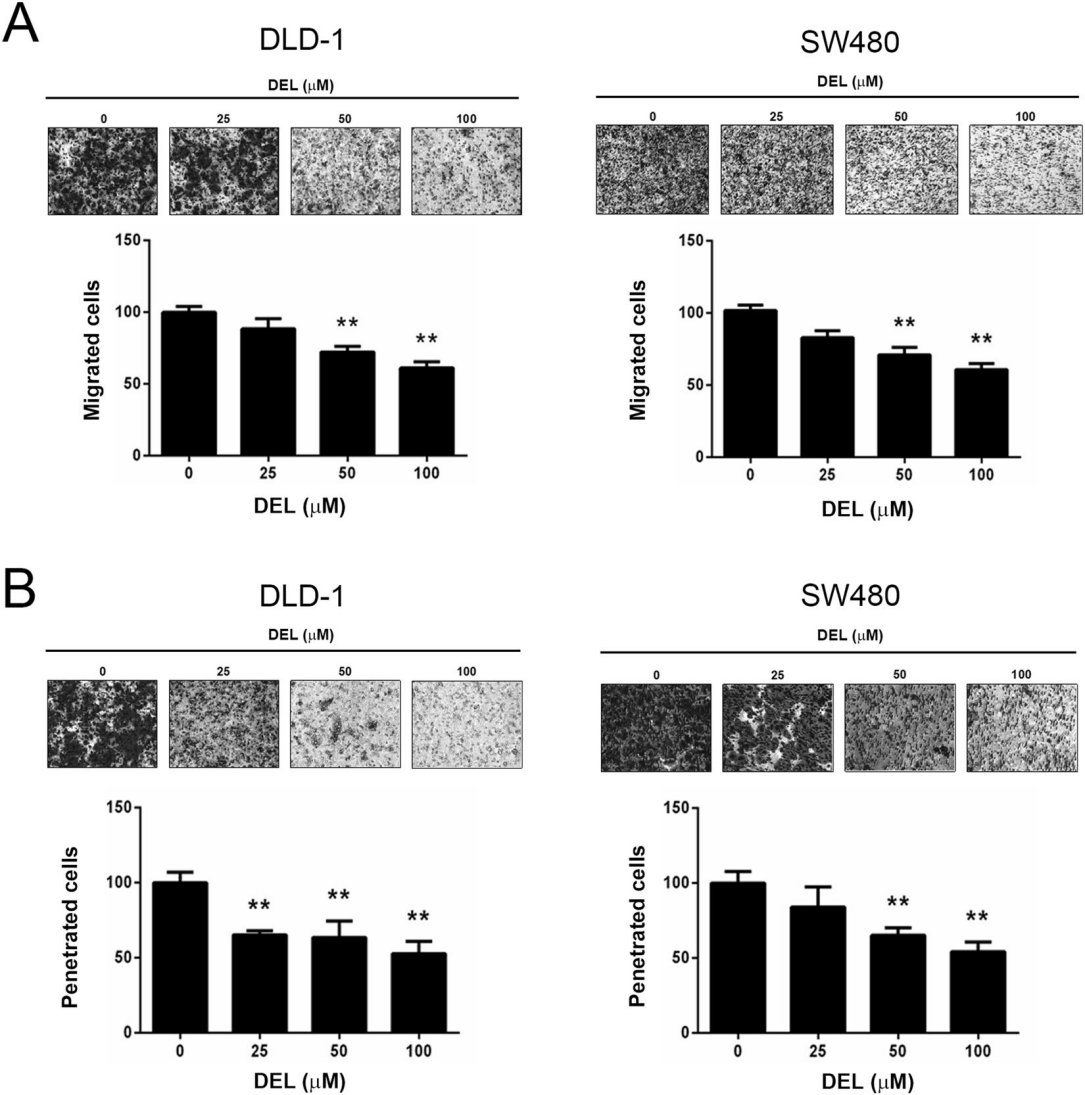
Delphinidin reduced cell migration and invasion of human CRC cells. Cells were cultured on membrane without or with Matrigel, treated with delphinidin (DEL) at serial concentrations for 24 h, and then subject to (**A**) transmigration assay or (**B**) invasion analysis. Quantitative data was acquired from three independent experiments and presented as mean ± standard deviation. **P < 0.01 as compared to the DMSO control (0 μM).

The potent migratory and invasive potential of cancer cells plays pivotal roles in metastasis. Thus, we investigated whether delphinidin could inhibit the motility and invasiveness of CRC cells. As shown in Fig. 3A, 50 and 100 μM delphinidin significantly (*P* < 0.01) and dose-dependently decreased the transmigration of DLD-1 and SW480 cells. Similarly, the number of invaded DLD-1 and SW480 cells was also dose-dependently decreased following delphinidin treatment (50 and 100 μM, *P* < 0.01; Fig. 3B). These results demonstrate that delphinidin inhibits the motility and invasiveness of CRC cells.

### Delphinidin suppressed the epithelial-mesenchymal transition of human CRC cells

**Figure 4:**
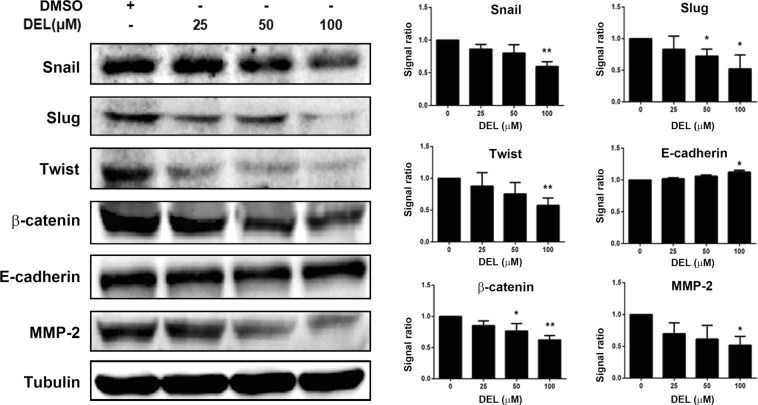
Delphinidin suppressed epithelial-to-mesenchymal transition in human CRC cells. Cells were treated with delphinidin (DEL) at serial concentrations for 24 h, then the cells were collected and lysed for the detection of epithelial and mesenchymal markers by using Western blotting. Quantitative data was acquired by using densitometric analysis from three independent experiments and presented as mean ± standard deviation. * and **P < 0.05 and *P* < 0.01 as compared to the DMSO control (0 μM).

EMT is the primary process that transforms solid epithelial tumors into mesenchymal metastatic cancer cells^27^. Therefore, we assessed whether delphinidin regulates the expression of EMT markers in CRC cells. As shown in Fig. 4, the key EMT inducers Snail, Slug, Twist, and β-catenin were significantly downregulated in DLD-1 cells following treatment with 100 μM delphinidin (*P* < 0.05). Similarly, delphinidin also decreased the expression of matrix metalloproteinase-2 (MMP-2), a proteinase that is highly associated with tumor metastasis^28^. In contrast, the expression of the epithelial marker E-cadherin was upregulated following delphinidin (100 µM) treatment (*P* < 0.05; Fig. 4).

### Delphinidin inhibited the integrin/FAK signaling cascade in DLD-1 cells

**Figure 5:**
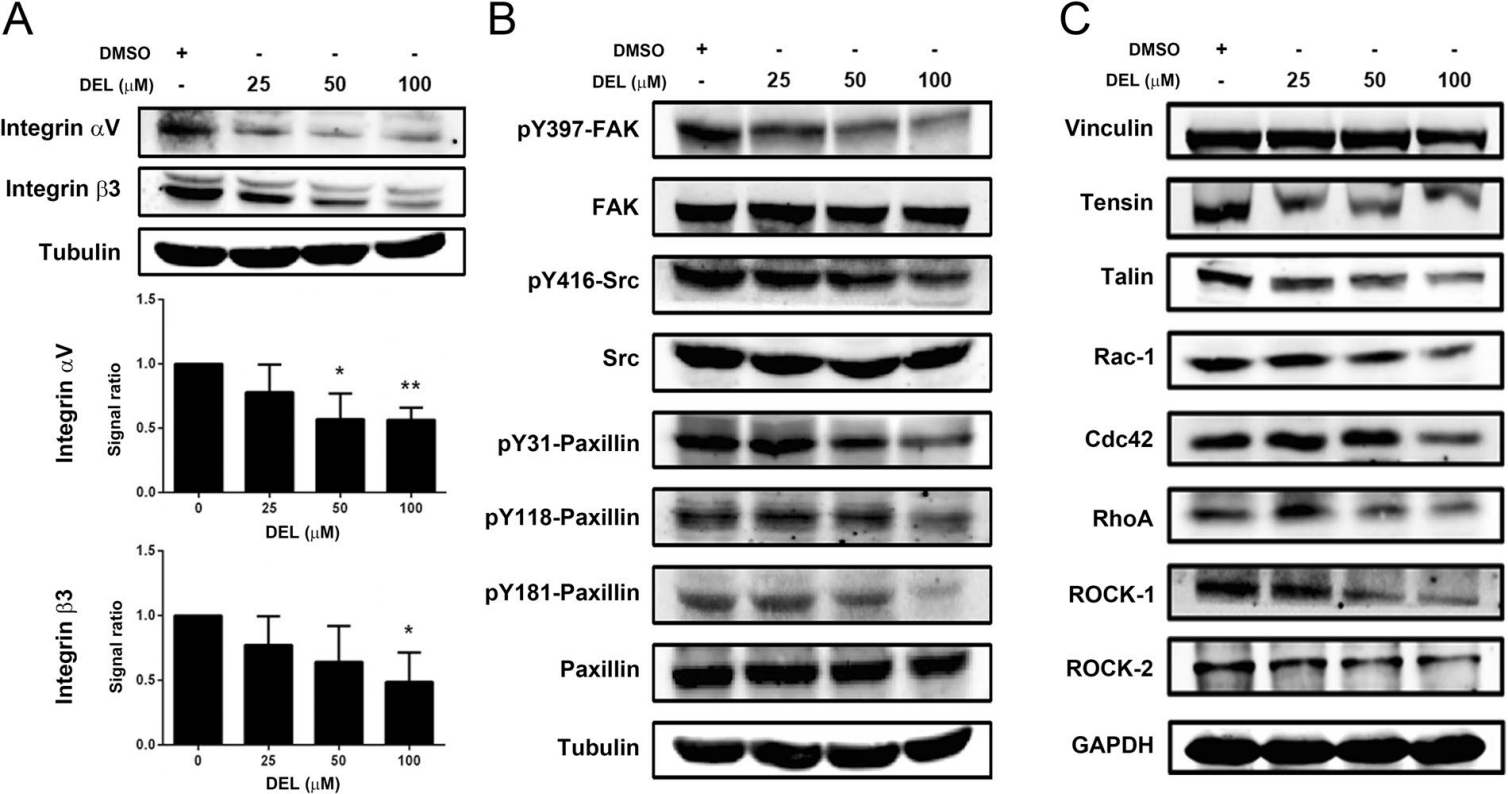
Delphinidin reduced integrin expression and inhibited FAK cascade in DLD-1 cells. Cells were treated with delphinidin (DEL) at serial concentrations for 24 h, then the cells were collected and lysed for the detection of (**A**) integrins, (**B**) FAK signaling components, and (**C**) integrin-associated adaptor proteins by using Western blotting. Quantitative data was acquired by using densitometric analysis from three independent experiments and presented as mean ± standard deviation. * and **P < 0.05 and *P* < 0.01 as compared to the DMSO control (0 μM).

Integrin and downstream FAK signaling constitute a pivotal pathway in the regulation of cell adhesion and migration^29,30^. Since delphinidin diminished the adhesion, motility, and invasiveness of CRC cells, the effects of delphinidin on integrin and FAK signaling were investigated. As shown in Fig. 5A, delphinidin significantly and dose-dependently (*P* < 0.05) reduced the expression of integrin αV and β3 in DLD-1 cells. In parallel to the reduced integrin expression, the downstream signaling components, including FAK phosphorylation (Tyr397), Src phosphorylation (Tyr416), and Paxillin phosphorylation (Tyr31, Tyr118, Tyr181), were diminished in response to delphinidin treatment (Fig. 5B). In addition, the intracellular integrin-associated adaptor proteins Tensin and Talin were also downregulated in DLD-1 cells following exposure to delphinidin (Fig. 5C). The FAK-mediated small GTPases Rac-1, Cdc42, and Rho A and the downstream effectors of Rho A, ROCK1, and ROCK2 were also decreased (Fig. 5C). These findings indicate that delphinidin not only downregulates integrin expression and integrin-associated adaptor proteins but also inhibits FAK activation and the downstream small GTPases and effectors.

### Delphinidin inhibited invasion and the integrin/FAK axis via the upregulation of miR-204-3p in DLD-1 cells

**Figure 6:**
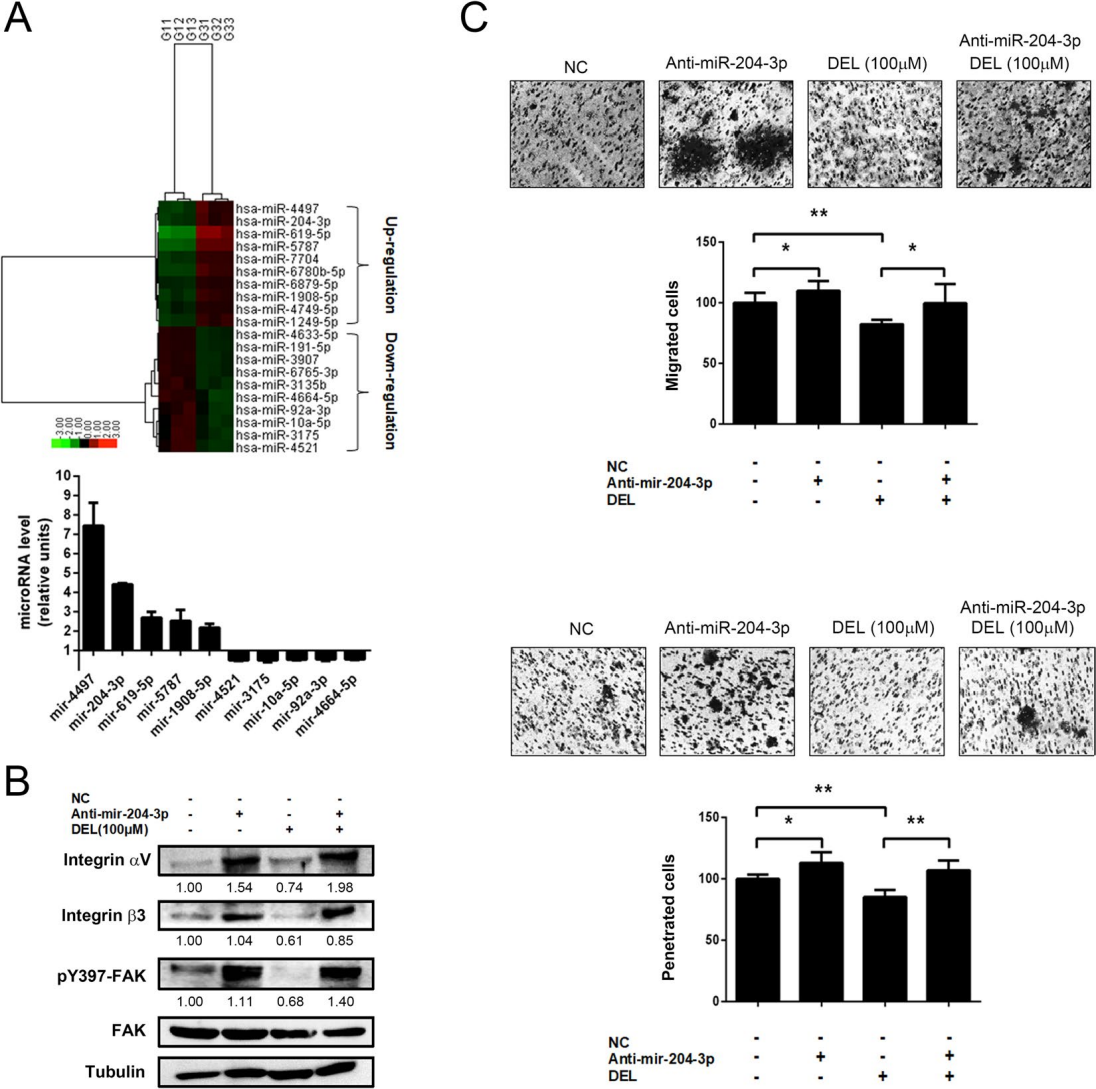
Involvement of miR-204-3p in the inhibited integrin/FAK cascade and the suppressed cell migration and invasion of human CRC cell DLD-1 in response to delphinidin. (**A**) Cells were treated with delphinidin (DEL) at 100 μM for 24 h, and then the cells were lysed for RNA extraction and microRNA expression profiling by using microarray analysis. MicroRNAs with up-regulation and down-regulation in response to delphinidin were labeled with red and green color, respectively. (**B**) Cells were transfected with control vector (NC) or anti-miR-204-3p, treated with delphinidin for 24 h, and then lysed for the assessment of integrin expression and FAK activation by Western blotting. (**C**) Cells were transfected with control vector or anti-miR-204-3p, treated with delphinidin for 24 h, and then subject to the transmigration and invasion assay. Quantitative data was acquired from three independent experiments and presented as mean ± standard deviation. * and **P < 0.05 and *P* < 0.01 as compared to the DMSO control (0 μM).

Accumulating evidence has shown that miRs play an important role in the motility and invasiveness of cancer cells and tumor metastasis^31,32^. Therefore, we evaluated whether delphinidin affects miR expression in CRC cells and explored the involvement of these miRs in the regulation of cell motility and integrin/FAK signaling. Using a microarray with 853 miRs, we observed that delphinidin treatment (100 μM for 24 h) altered the miR expression profile in DLD-1 cells. We identified 24 upregulated and 22 downregulated miRs with an expression ratio (delphinidin/control) log_2_ > 0.585 and < −0.585, respectively. The top ten most significantly up- and down-regulated miRs in response to delphinidin are presented in Fig. 6A (upper panel). Densitometric analysis showed that the expression of miR-204-3p was obviously upregulated, at ~4.5-fold (*P* < 0.01, Fig. 6A, lower panel). miR-204-3p has been reported to be involved in the invasion of renal cell carcinoma^33^. Therefore, the role of miR-204-3p in delphinidin-inhibited integrin/FAK signaling, transmigration, and invasion of DLD-1 cells was analyzed. As shown in Fig. 6B, the delphinidin-induced decreases in integrin αV/β3 levels and FAK phosphorylation (Tyr397) were restored in DLD-1 cells transfected with an Anti-mir-204-3p as compared to the values in cells transfected with a control vector (NC). Similarly, the delphinidin-induced inhibition of transmigration and invasion were also recovered in DLD-1 cells transfected with Anti-mir-204-3p when compared to control vector-transfected cells (NC, Fig. 6C, upper and lower panels). Collectively, these results show that delphinidin regulates miR expression in DLD-1 cells and that mir-204-3p plays an important role in the suppression of integrin/FAK signaling and the migration/invasion induced in response to delphinidin.

### Delphinidin inhibited the *in vivo* metastasis of DLD-1 colon cancer cells

**Figure 7:**
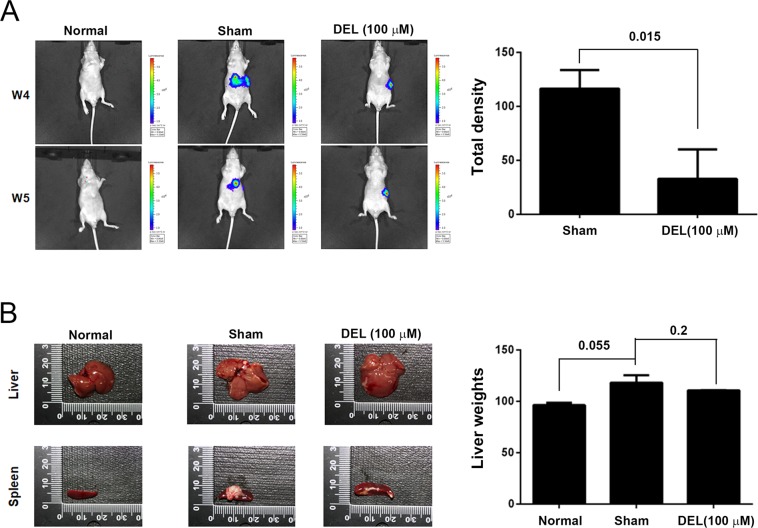
Delphinidin *in vivo* attenuated the metastasis of human CRC cell DLD-1 in xenograft mice. DLD-1 cells stably expressing luciferase were intraperitoneally injected into mice, and (**A**) the metastasized DLD-1 cells were detected by using IVIS image system after two weeks, then (**B**) the mice were sacrificed to acquire liver samples for phenotyping and weighing. Quantitative data was acquired by using photodensitometric analysis from three independent experiments and presented as mean ± standard deviation. *P* values as compared to normal or sham control were indicated.

Based on the observation that delphinidin treatment suppressed the migration and invasion of CRC cells *in vitro*, we further investigated whether delphinidin inhibits the metastasis of a highly invasive CRC cell line (DLD-1) when compared to SW480 and SW620 cells. As shown in Fig. 7A, the metastasis of delphinidin-treated DLD-1 cells (100 μM for 24 h) to the lung was significantly reduced as compared to that of DMSO-treated DLD-1 cells (*P* < 0.05). In addition, the weights of the livers from DLD-1 model mice treated with DMSO or delphinidin (120.6 ± 5.6 mg and 110.6 ± 0.8 mg, respectively) were statistically the same (Fig. 7B, *P* = 0.2). These observations show that delphinidin treatment significantly attenuated the *in vivo* metastatic ability of DLD-1 cells but did not affect liver and spleen weights.

## Discussion

Cell motility, EMT, and carcinogenicity are closely associated with the progression and metastasis of CRC, and early metastasis is the main cause of mortality in patients with CRC. Here, we found that delphinidin inhibited the adhesion, colony formation, motility, and invasion of CRC cells, which may be attributed to the inhibition of EMT, suppression of integrin/FAK signaling, and upregulation of miR-204-3p. These findings suggest that delphinidin has promising anti-metastatic potential in CRC.

Previous reports have demonstrated that several phenolic acids, including anthocyanins, protocatechuic acid (PCA), syringic acid, vanillic acid, phloroglucinol aldehyde, phloroglucinol acid, and gallic acid (GA), are metabolites of anthocyanins^34^, and the interplay between anthocyanins and the gastrointestinal microbiota plays a central role in producing these metabolites^35^. De Ferrars *et al*. analyzed the metabolite profile of cyanidin-3-glucoside in humans using high-sensitivity mass spectrometry, and identified 17 metabolites in serum, urine, and feces^36^, indicating that anthocyanins can be absorbed and metabolized by humans and the resulting metabolites are widely distributed in body fluids. In addition, a recent study reported that GA is a degradation product of delphinidin and both delphinidin and GA have potent biological activities, including anti-cancer and anti-inflammatory activities^37–39^. In our *in vitro* experiments, delphinidin treatments were conducted in a neutral pH condition; therefore, delphinidin may be partially transformed to the degradation products such as GA. Our *in vitro* findings clearly show that delphinidin has anti-metastatic effects on CRC cells. Our *in vivo* findings using a xenograft model also show that delphinidin attenuates the metastatic ability of xenografted DLD-1 cells in mice. Taken together, these observations indicate that delphinidin as well as its metabolites, such as GA, may directly and/or synergistically exert anti-metastatic effects on CRC cells.

Integrins are well-characterized cell surface receptors that are composed of non-covalent, heterodimeric complexes with an α subunit and a β subunit. The major signaling pathway downstream of integrin is the FAK cascade, which has been widely reported to be involved in EMT, a process that leads to the invasion and metastasis of various tumors^40^. During EMT, dynamic changes in the cytoskeleton lead to a loss of cell-cell contacts and epithelial cell polarity, accompanied with enhanced cell motility. Thus, potent EMT inducers, including Snail, Slug, Twist, and ZEB2, have been implicated in tumor progression and metastasis^41,42^. Snail and ZEB2 have also been reported to affect cell-matrix adhesion by modulating integrins and basement membrane proteins^43^. In this study, we found that delphinidin downregulated the expression of the EMT inducers Snail, Slug, Twist, and β-catenin in DLD-1 cells. In addition, delphinidin also decreased the expression of integrin and its adaptor proteins Vinculin, Talin, and Tensin and suppressed proteins in the FAK signaling cascade, including Src, Paxillin, Rac-1, Cdc42, Rho A, and ROCK-1/2. These findings indicate that delphinidin exerts both anti-EMT and anti-metastatic activities in CRC cells through inhibition of the integrin/FAK cascade.

miRs have been shown to play important roles in carcinogenesis, tumor progression, and metastasis^44–46^. Through the miRNA array analysis, we demonstrated that delphinidin can alter the miR expression profile in DLD-1 cells; 24 miRs upregulated and 22 miRs were downregulated (data not shown). The biological functions of several of the identified miRs have been investigated; however, most of the miRs that were significantly changed in response to delphinidin are first found to be associated with the motility and invasiveness of CRC cells. For example, miR-4497 has been reported as a tumor suppressor in laryngeal squamous cell carcinoma that functions through inhibition of Gastrulation Brain Homeobox 2, a homeobox gene involved in the normal development of rhombomeres^47^. In addition, miR-204-3p was shown to attenuate high glucose-induced apoptosis of podocytes by suppressing Bradykinin B2 Receptor^48^. Finally, overexpression of miR-92a-3p in the peripheral blood mononuclear cells of patients with chronic lymphocytic leukemia has been reported as a potential independent biomarker associated with prolonged overall survival^49^. However, further investigation is required to determine the roles of these miRs in regard to the anti-metastatic activity of delphinidin in CRC cells.

In conclusion, our results show that miR-204-3p is upregulated in response to delphinidin and that miR-204-3p plays an important role in the regulation of integrin/FAK signaling and the invasion of DLD-1 cells. These findings reveal that delphinidin inhibits the motility and invasiveness of CRC cells by regulating the miR-204-3p-mediated integrin/FAK cascade. *In vivo* metastasis analysis also showed similar inhibitory effects of delphinidin on DLD-1 cells. Taken together, the study results suggest that miR-204-3p may be a potential molecular target for CRC therapy.

## Methods

### Chemicals and antibodies

All general chemicals were purchased from Sigma-Aldrich (St. Louis, MO, USA), including delphinidin (≥95%, no. 43725); Giemsa; crystal violet; 2-propanol; 3-(4,5-dimethylthiazol-2-yl)-2,5-diphenyltetrazolium bromide (MTT); 1-butanol; dimethyl sulfoxide (DMSO); phosphate-buffered saline (PBS); sodium chloride; sodium dodecyl sulfate (SDS); Tris-HCl; type I collagen (no. C7661); and trypsin/EDTA. Primary antibodies specific binding to human Snail (SC-393172), Slug (SC-166902), Twist (SC-81417), E-cadherin (SC-71007), β-catenin (SC-59737), glyceraldehyde 3-phosphate dehydrogenase (GAPDH, SC-47724), matrix metalloproteinase-2 (MMP-2, SC-13594), tubulin (SC-134237), integrin αV (SC-376156), integrin β3 (SC-365679), focal adhesion kinase (FAK, SC-271126), phospho-FAK (pY397-FAK, SC-81493), Src (SC-32789), phospho-Src (pY416-Src, SC-24621, CS#2101), paxillin (SC-136297), and phospho-paxillin (pY118-Paxillin, SC-365020), and peroxidase-conjugated secondary antibodies specific binding to mouse IgG and rabbit IgG were purchased from Santa Cruz Biotechnology (Santa Cruz, CA, USA). Antibodies against human phospho-paxillin (pY31-Paxillin and pY181-Paxillin) were purchased from BioSource International (Camarillo, CA, USA).

### Cell culture and treatments

The human colon cancer cell lines DLD-1, SW480, and SW620 were acquired from ATCC and maintained in RPMI-1640 medium and Dulbecco’s modified Eagle’s medium (DMEM) (Sigma-Aldrich) containing 10% v/v fetal bovine serum (FBS; Biological Industries, Cromwell, CT, USA) and incubated at 37 °C with 5% CO_2_. For delphinidin treatment, cells reached 80% confluence were incubated with serial concentrations of delphinidin (20–100 μM). DMSO alone (final concentration, 0.1%) was used as the solvent control (presented as 0 μM delphinidin). After the 24 hour (h)-treatment, the cells were collected, washed with PBS, and used for the following experiments.

### Cell viability assay

Viable cells were assessed by using the MTT assay. In brief, 2 × 10^5^ cells were seeded into a 24-well plate containing 10% v/v FBS culture medium for 24 h to allow cells attached to the plate, then the attached cells were treated with serial concentrations of delphinidin (20–100 μM) for 24 h. The treated cells were washed with PBS twice and incubated with MTT reagent (5 mg/mL; Sigma-Aldrich) at 37 °C for 2 h. After removing the supernatant, isopropanol was added and the solubilized formazan was determined by measuring the absorbance at 563 nm using a spectrophotometer (U-2900; Hitachi, Tokyo, Japan). The percentage of viable cells was estimated by comparison to the absorbance of DMSO-treated cells. Three independent experiments were performed for statistical analysis.

### Colony formation assay

Cells were resuspended in the agarose medium consisting RPMI-1640 medium, 10% v/v FBS, 0.3% w/v agarose, and delphinidin, and plated in a 6-well plate pre-coated with agarose (RPMI medium containing 10% v/v FBS and 0.6% w/v agarose). After incubated in a humidified atmosphere at 37 °C containing 5% CO_2_ for 7 days, the cells were fixed, stained with crystal violet, and then photographed under a microscope (Nikon Eclipse TE2000-U equipped with a Nikon Digital Camera DXM1200; Nikon, Japan). Colonies greater than 0.1 mm in diameter were counted.

### Cell adhesion assay

Cells were incubated with serial concentrations of delphinidin for 24 h, and then transferred to 12-well plates coated with type I collagen (10^5^ cells per well). After incubated at 37 °C for 8 h, non-adherent cells were removed by washing with PBS. Then, the adhered cells were visualized with crystal violet staining, photographed, and quantitated using a cell counter.

### Transmigration and invasion assays

The cell migratory ability was assessed by transmigration assay. In brief, cells pretreated with serial concentrations of delphinidin for 24 h were seeded onto 24-well Millicell® Hanging Cell Culture inserts (8 μm pore size; Millipore, Bedford, MA, USA). FBS (20%) was added to the lower compartment as a chemoattractant, and then the cells were incubated at 37 °C for 12 h. The cells that transmigrated to the lower surface of the insert were fixed with 4% paraformaldehyde, visualized using Giemsa staining (Sigma-Aldrich), and then photographed. The numbers of transmigrated cells in five random fields under the microscope were counted for the quantitative analysis.

The cell invasiveness was assessed by invasion assay. In brief, cells were seeded onto the insert which was pre-coated with 100 μL of Matrigel (20× diluted with PBS; BD Biosciences, San Jose, CA, USA), and then incubated for 16 h. Cells that invaded the lower surface of the insert were visualized using Giemsa staining and quantitated as described in the transmigration assay.

### Western blotting

After treatment, the cells were detached by trypsinization, collected by centrifugation, and then lysed in the PBS lysis buffer containing protease and phosphatase inhibitor cocktail (Sigma-Aldrich). The cell lysates were centrifuged at 20,000 x g at 4 °C for 10 min, and then the supernatant was collected and used as a crude extract. The protein concentration in the extracts was assessed by Bradford protein assay (Bio-Rad Laboratories, Hercules, CA, USA). Extracts containing equal protein (30 µg) were separated by electrophoresis on an SDS-polyacrylamide gel. The electrophoresed proteins were transferred to a nitrocellulose membrane (PROTRAN BA85, 0.45 μm; Sigma) with a Transphor Unit (Bio-Rad Laboratories, Hercules, CA, USA). The transferred membrane was blocked with 1% (w/v) BSA in PBS followed by a 1-h incubation with the primary antibodies and then the secondary antibodies. The signal was developed with ECL chemiluminescence reagent (SuperSignal West Dura HRP Detection Kit; Pierce Biotechnology, Rockford, IL, USA), and the signals were acquired and quantitated with an image analysis system (Fujifilm, Tokyo, Japan).

### Microarray analysis for miR profiling

DLD-1 cells treated with 100 μM delphinidin or 0.1% DMSO for 24 h were used for the miRNA microarray analysis. In brief, total RNA from DLD-1 cells was extracted using the miRNeasy mini kit (QIAGEN, Mainz, Germany) and labeled with Cy3 during reverse transcription to cDNA using an Agilent miRNA Labeling Kit (Agilent, UK) and Spike Kit (Agilent, UK). The labeled cDNAs were hybridized (in duplicate) to Human miRNA Microarray, Release 19.0, 8 × 60 K (v19) microarray slides (Agilent, UK) according to the microRNA Hybridization Kit protocol (Agilent, UK) and scanned using a Nimblegen MS200 array scanner. Data were normalized using the Plier algorithm and were log transformed and analyzed using GeneSpring GX 13.0 (Agilent, UK). miRs with *P* values less than 0.05 and fold changes >2 or <−2 between control (DMSO) and delphinidin-treated cells were considered significantly changed.

### miR transfection

The Anti-miR-204-3p and corresponding negative control (NC) vectors were obtained from System Biosciences (Palo Alto, CA, USA). DLD-1 cells were transfected with Anti-miR-204-3p or Anti-miR-NC using Lipofectamine 2000 (Invitrogen, Carlsbad, CA, USA) according to the manufacturer’s instructions.

### Xenograft animal model and cancer cell imaging

Male Balb/c nude mice were obtained from the National Laboratory Animal Center of Taiwan (Taipei City, Taiwan) and maintained under the supervision of the Institutional Animal Care and Use Committee (IACUC) of Chung Shan Medical University. All the protocols for animal experiments have been approved by the IACUC (No. 1947), and all the experiments were performed in accordance with the approved guidelines. After a 1-week acclimation period, the mice were randomly divided into the following three groups (n = 6 per group): Normal (no cancer cell implantation), Sham (implantation of cancer cells treated with 0.1% DMSO/PBS for 24 h), and Delphinidin (implantation of cancer cells treated with 100 μM delphinidin for 24 h). Cancer cell implantation was performed by intraperitoneal injection of luciferase-transfected DLD-1 cells (2 × 10^6^ cells in 50 μL of PBS), and the animals were maintained for two weeks. After the maintenance period, the mice were anesthetized and intraperitoneally injected with luciferin (at 150 mg/kg in 100 μL). Images were captured at 20 min after injection using an IVIS-200 Imaging System (Xenogen, Alameda, CA, USA) and processed using Living Image software (Xenogen) by region-of-interest analysis of the total photons per second for each tumor, with background subtraction. After the image analysis was completed, the mice were sacrificed to examine the organs.

### Statistical analysis

The data represent the mean ± SD of three independent experiments, except where indicated. Student’s *t* test was used to analyze the significance of differences. *P* values less than 0.05 were considered statistically significant.

## Supplementary information

Supplementary information
