# Supplementary material for: Dietary delphinidin inhibits human colorectal cancer metastasis associating with upregulation of miR-204-3p and suppression of the integrin/FAK axis
Source: Sci Rep. 2019 Dec 12;9:18954. doi: 10.1038/s41598-019-55505-z (PMC6908670; doi:10.1038/s41598-019-55505-z)

Supplementary information for

Dietary delphinidin inhibits human colorectal cancer metastasis associating with upregulation of miR-204-3p and suppression of the integrin/FAK axis

Chi-Chou Huang[1,2#], Chia-Hung Hung[3#], Tung-Wei Hung[4,5], Yi-Chieh Lin[3], Chau-Jong Wang[3,6*], Shao-Hsuan Kao[3,6*],

[1]Department of Colorectal Surgery, Chung Shan Medical University Hospital, Taichung, Taiwan.

[2]School of Medicine, Chung Shan Medical University, Taichung, Taiwan.

[3]Institute of Biochemistry, Microbiology, and Immunology, College of Medicine, Chung Shan Medical University, Taichung, Taiwan.

[4]Institute of Medicine, Chung Shan Medical University, Taichung, Taiwan.

[5]Division of Nephrology, Department of Internal Medicine, Chung Shan Medical University Hospital, Taichung, Taiwan.

[6]Clinical Laboratory, Chung Shan Medical University Hospital, Taichung 402, Taiwan

[#] Chi-Chou Huang and Chia-Hung Hung contributed equally to this work.

Supplementary data

Figure 4

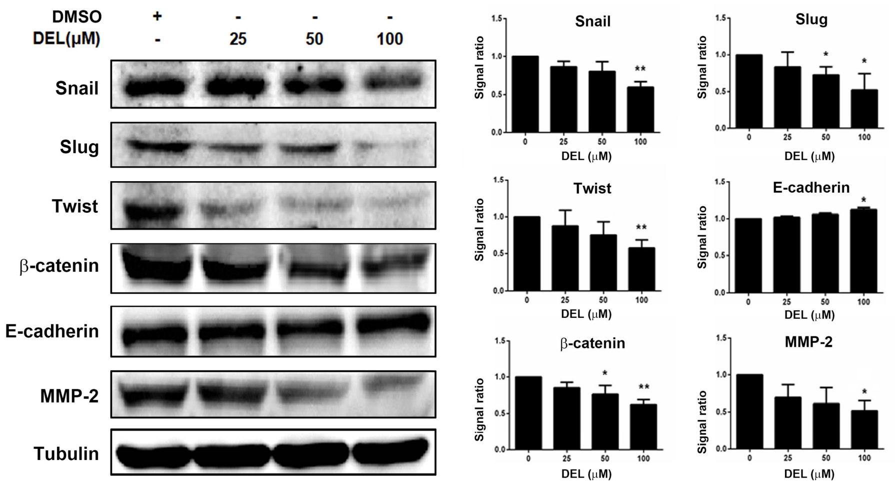

Snail

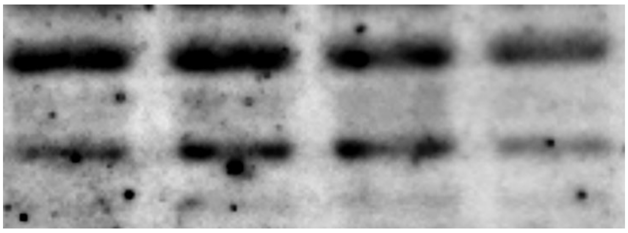

Slug

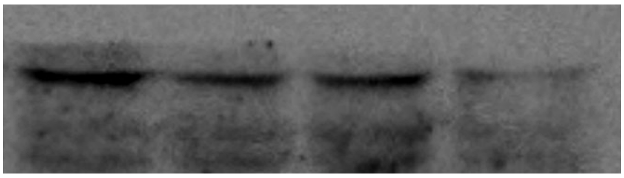

Twist

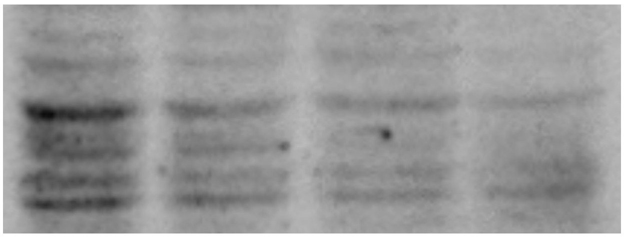

β-catenin

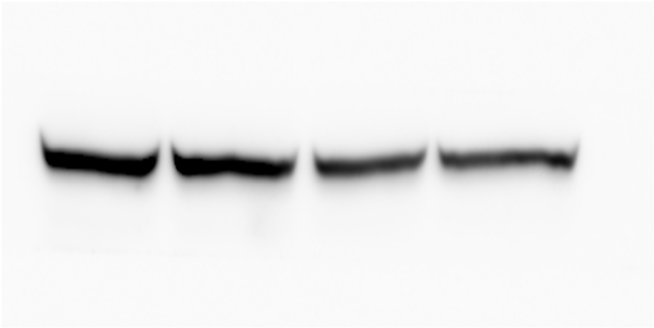

E-cadherin

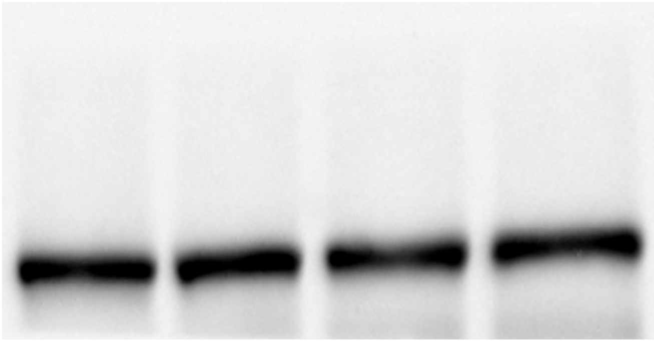

MMP-2

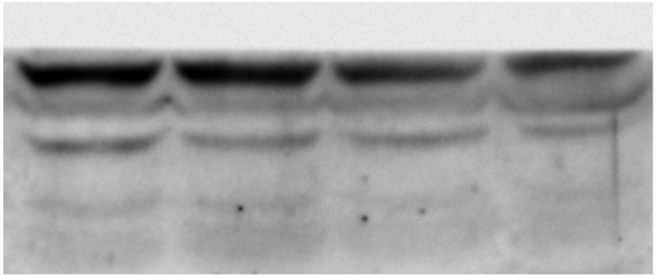

edge of
cutted membrane

Tubulin

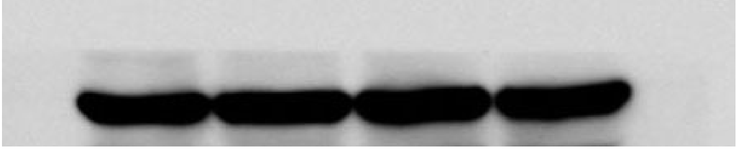

Figure 5

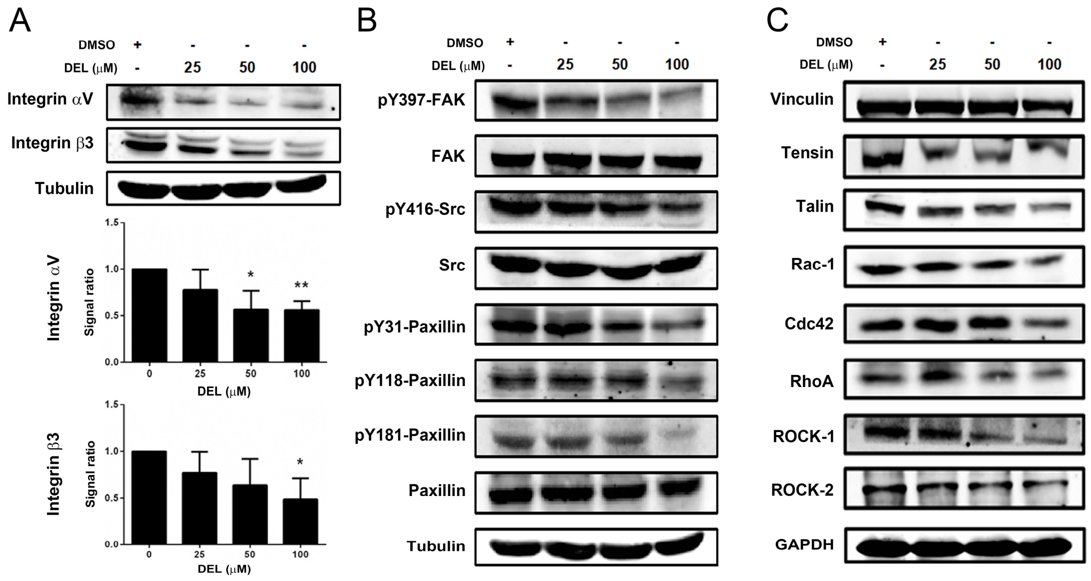

Figure 5A

Integrin αV

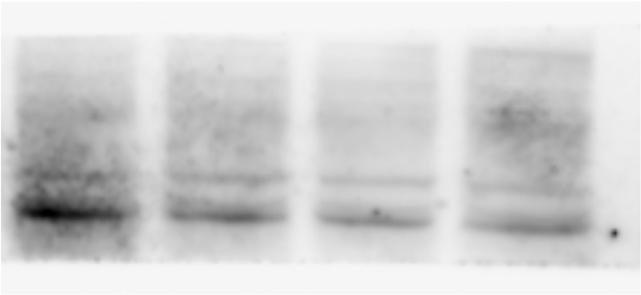

Integrin β3

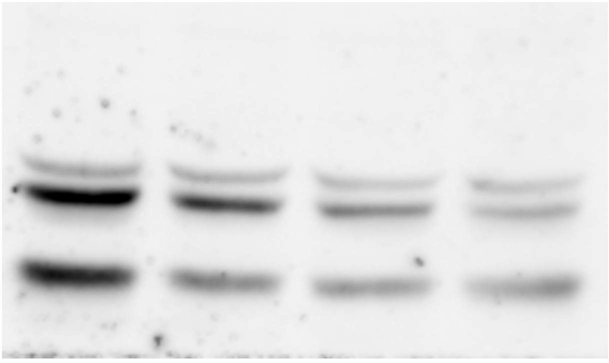

Tubulin

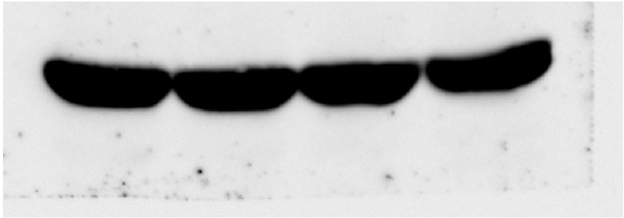

Figure 5B

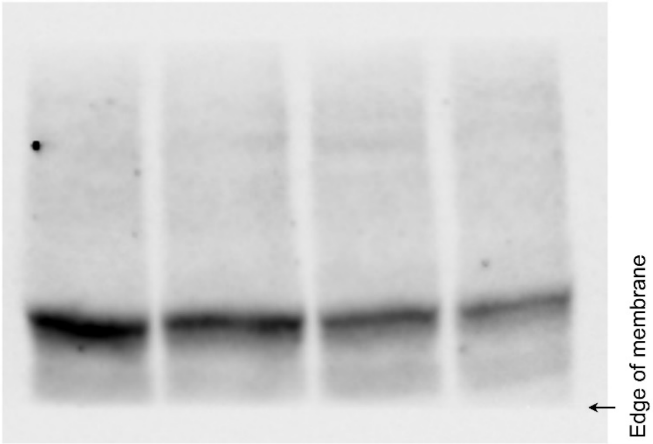

FAK (total)

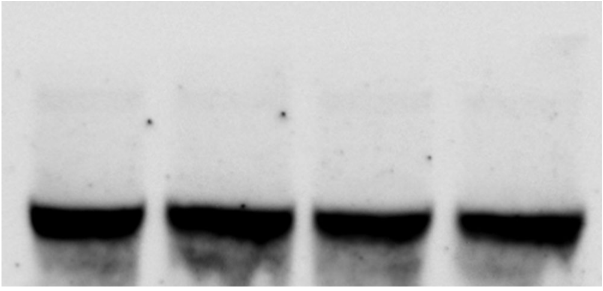

pY416-Src

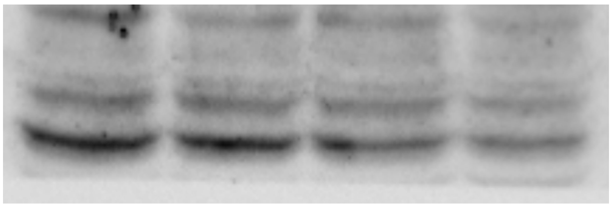

Src (total)

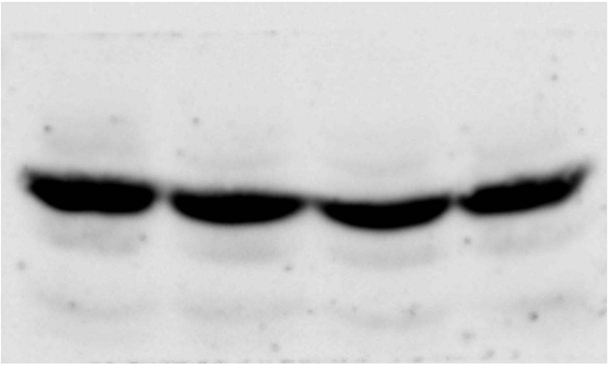

pY31-Paxillin

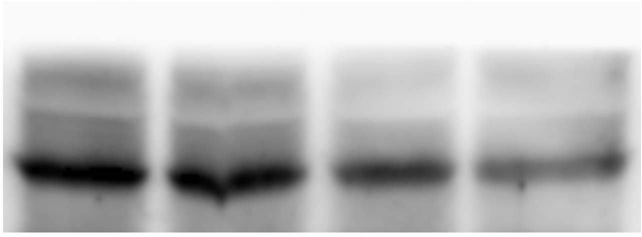

pY118-Paxillin

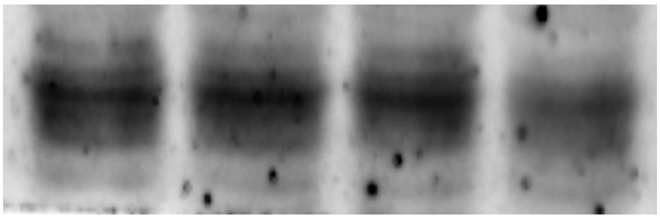

pY181-Paxillin

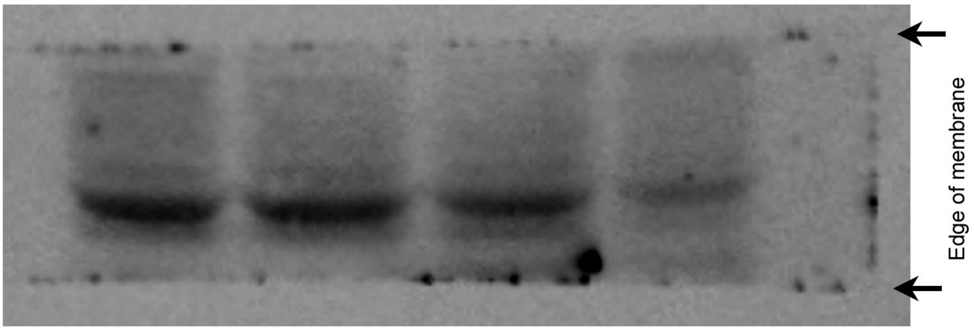

Paxillin (total)

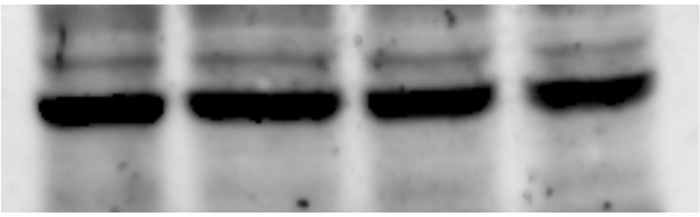

Tubulin

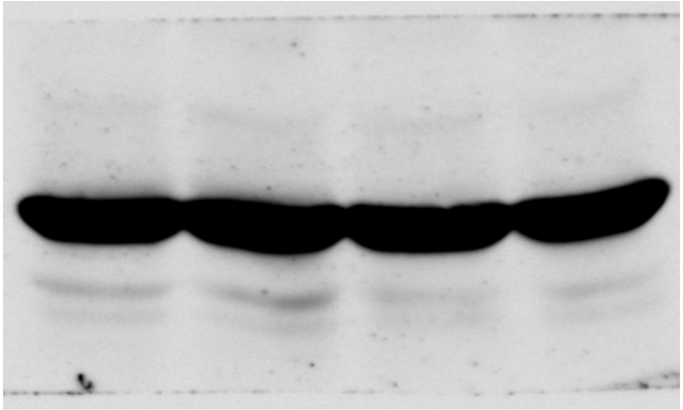

Figure 5C

Vinculin

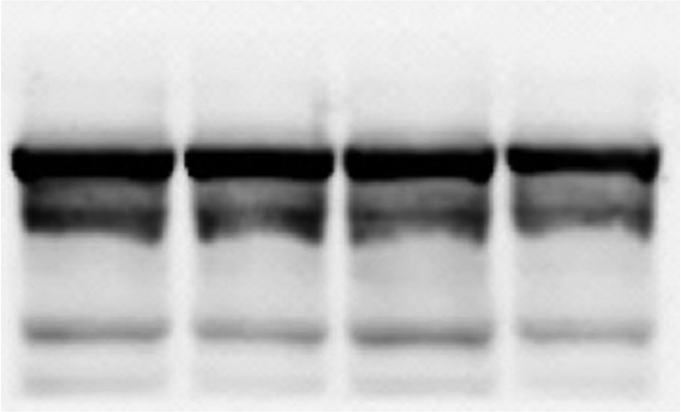

Tensin

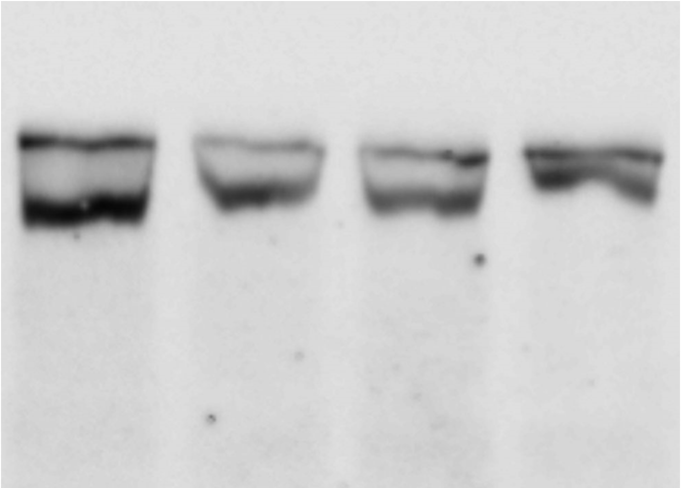

Talin

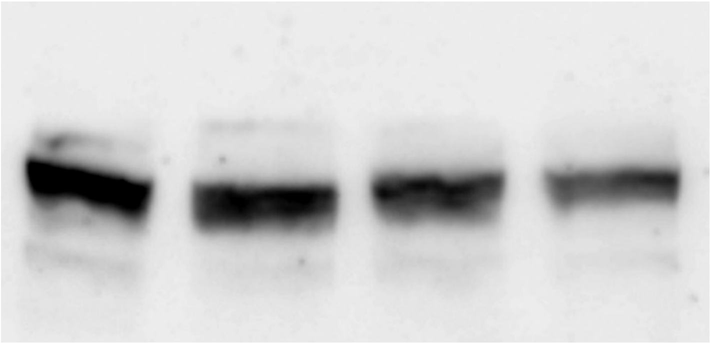

Rac1

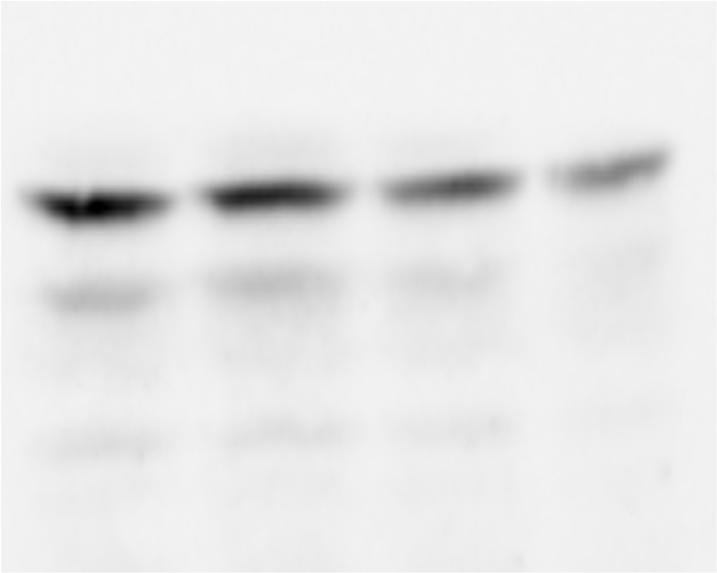

Cdc42

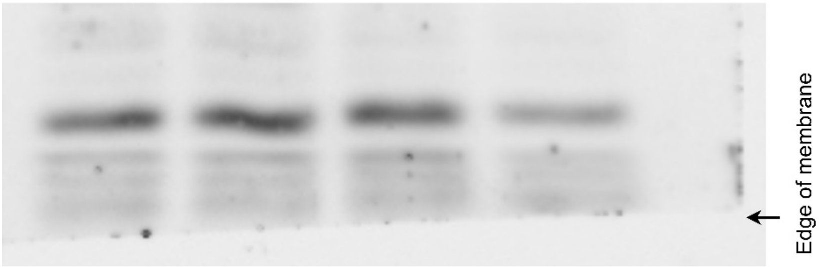

RhoA

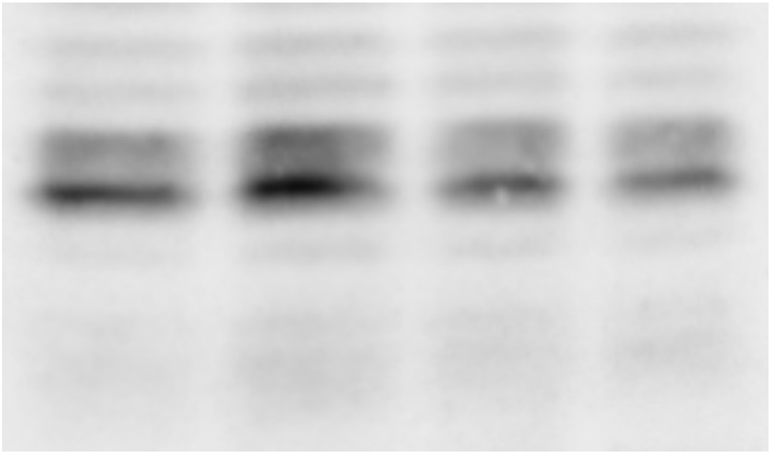

ROCK-1

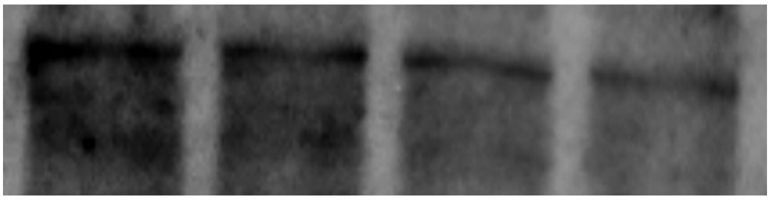

ROCK-2

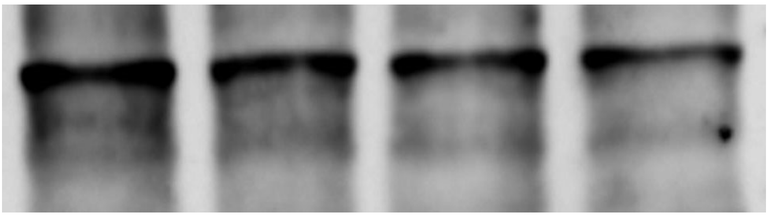

GAPDH

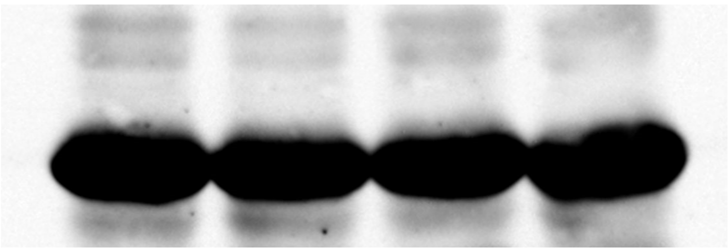

Figure 6

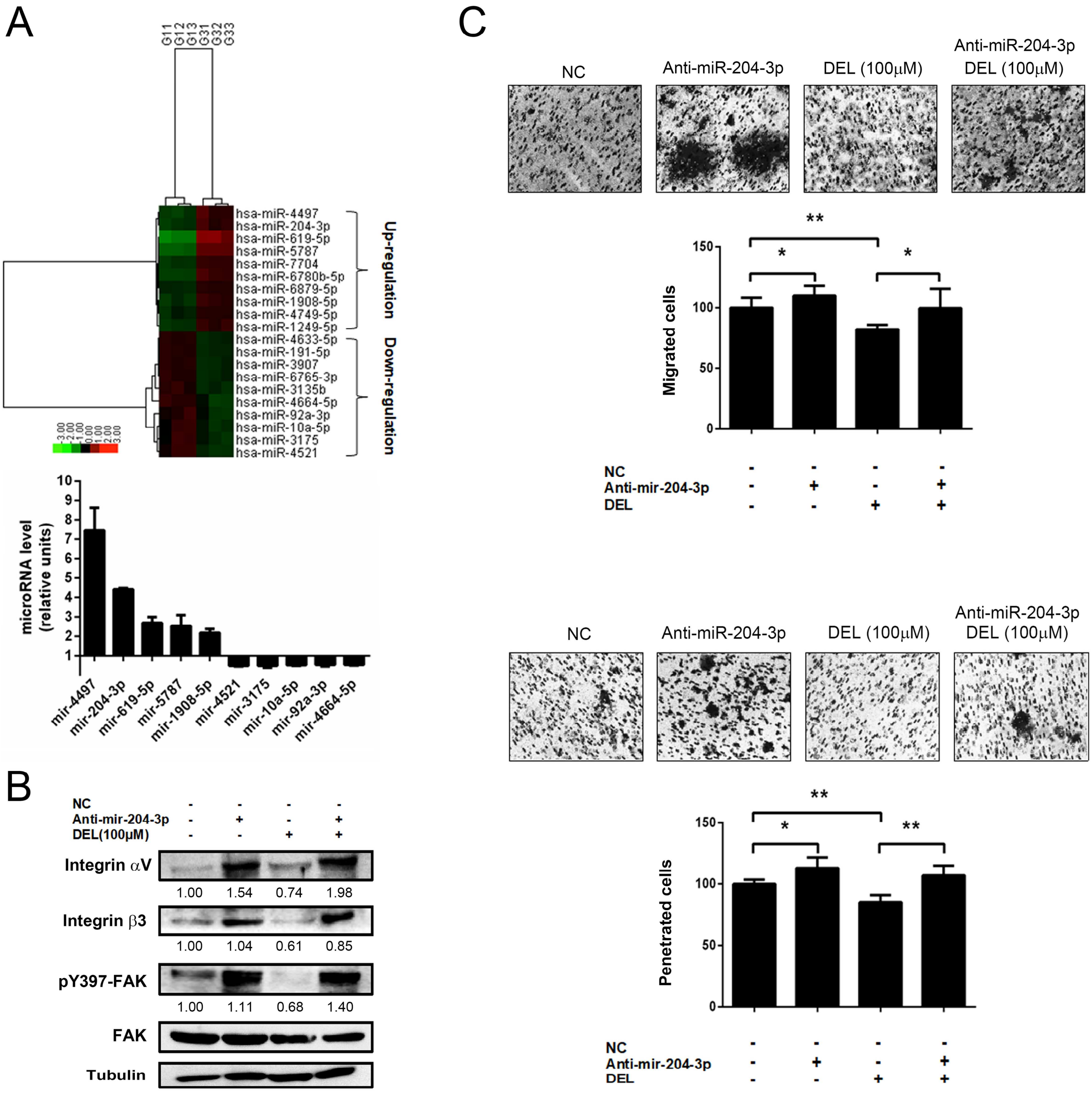

Figure 6B

Integrin αV

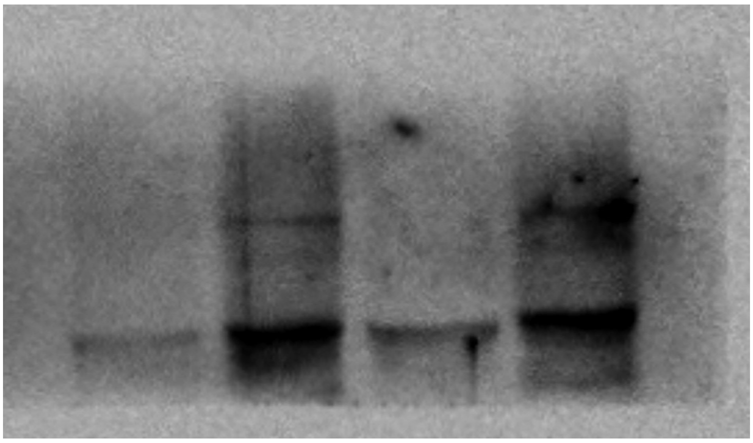

Integrin β3

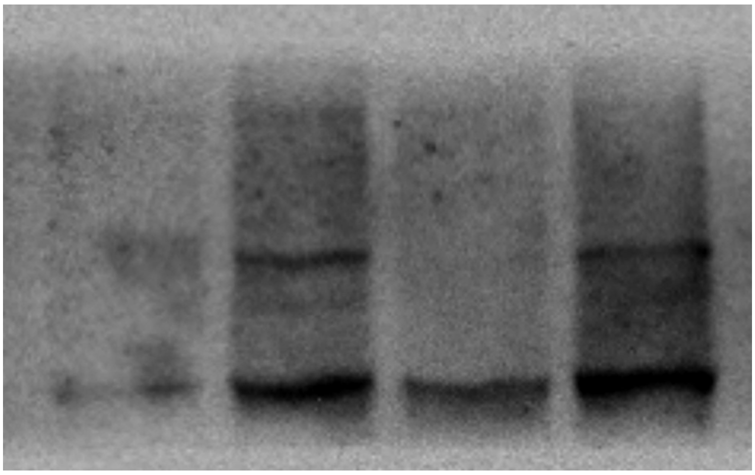

pY397-FAK

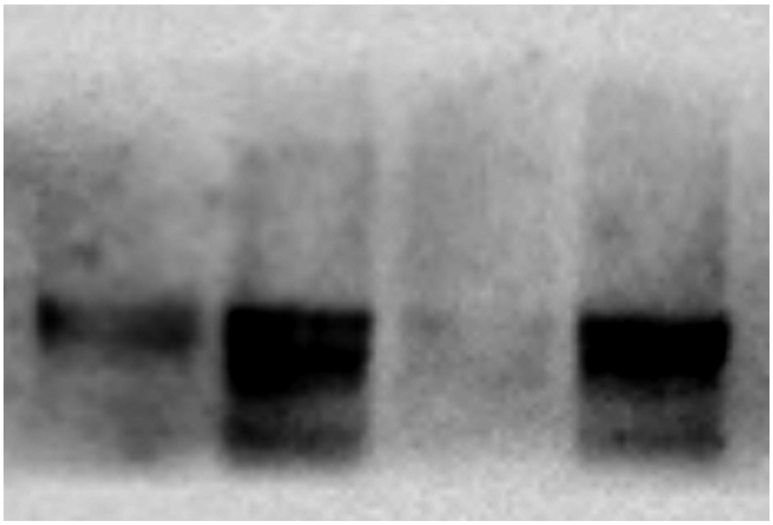

FAK (total)

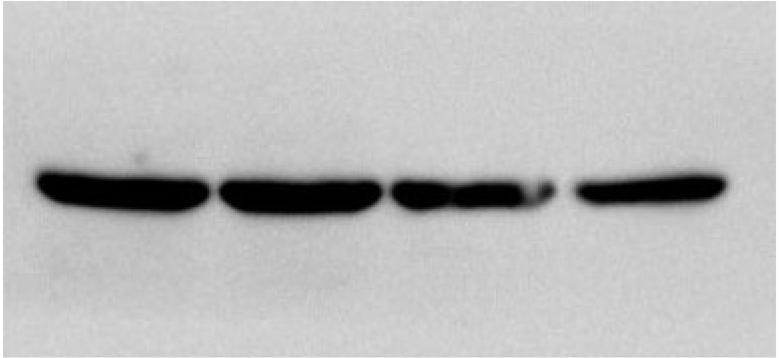

Tubulin

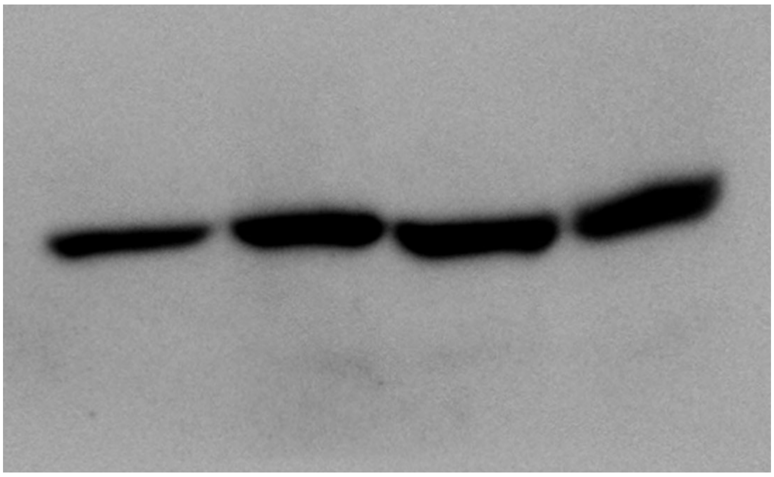

Supplement: Supplementary file 1 — Supplementary information [file 41598_2019_55505_MOESM1_ESM.pdf]
